# Retraction: Mitigation of NaCl Stress by Arbuscular Mycorrhizal Fungi through the Modulation of Osmolytes, Antioxidants and Secondary Metabolites in Mustard (*Brassica juncea* L.) Plants

**DOI:** 10.3389/fpls.2016.02040

**Published:** 2016-12-23

**Authors:** Frontiers Editorial Office

The authors have requested the retraction of the 4 July 2016 cited above. Following publication, concerns were raised regarding the scientific validity of the article. This was due to an undisclosed error in the experimental design (such as the presence of host tomato plants) and crucial experiments missing from the manuscript which did not support the hypotheses put forward in the paper. This retraction was approved by the Field Chief Editor and the Specialty Chief Editor of *Frontiers in Plant Science*.

The authors regret any inconvenience this may have caused to the reviewers, editors, and readers of *Frontiers in Plant Science*.

